# Establishment of an AAV Reverse Infection-Based Array

**DOI:** 10.1371/journal.pone.0013479

**Published:** 2010-10-19

**Authors:** Xiaoyan Dong, Wenhong Tian, Gang Wang, Zheyue Dong, Wei Shen, Gang Zheng, Xiaobing Wu, Jinglun Xue, Yue Wang, Jinzhong Chen

**Affiliations:** 1 State Key Laboratory of Genetic Engineering, Institute of Genetics, School of Life Science, Fudan University, Shanghai, China; 2 Beijing FivePlus Molecular Medicine Institute, Beijing, China; 3 State Key Laboratory for Molecular Virology and Genetic Engineering, National Institute for Viral Disease Control and Prevention, Chinese Center for Disease Control and Prevention, Beijing, China; Yale Medical School, United States of America

## Abstract

**Background:**

The development of a convenient high-throughput gene transduction approach is critical for biological screening. Adeno-associated virus (AAV) vectors are broadly used in gene therapy studies, yet their applications in *in vitro* high-throughput gene transduction are limited.

**Principal Findings:**

We established an AAV reverse infection (RI)-based method in which cells were transduced by quantified recombinant AAVs (rAAVs) pre-coated onto 96-well plates. The number of pre-coated rAAV particles and number of cells loaded per well, as well as the temperature stability of the rAAVs on the plates, were evaluated. As the first application of this method, six serotypes or hybrid serotypes of rAAVs (AAV1, AAV2, AAV5/5, AAV8, AAV25 m, AAV28 m) were compared for their transduction efficiencies using various cell lines, including BHK21, HEK293, BEAS-2BS, HeLaS3, Huh7, Hepa1-6, and A549. AAV2 and AAV1 displayed high transduction efficiency; thus, they were deemed to be suitable candidate vectors for the RI-based array. We next evaluated the impact of sodium butyrate (NaB) treatment on rAAV vector-mediated reporter gene expression and found it was significantly enhanced, suggesting that our system reflected the biological response of target cells to specific treatments.

**Conclusions/Significance:**

Our study provides a novel method for establishing a highly efficient gene transduction array that may be developed into a platform for cell biological assays.

## Introduction

High-throughput gene transduction methods are needed for gene function studies and drug discovery. Recently, reverse transfection or reverse infection (RI) approaches have been established by several groups [1], [2], [3], which appear to be promising for the large-scale analysis of gene function. Different from conventional transfection or infection of living cells using DNA (or RNA) or a viral vector, reverse transfection or infection requires immobilization of the DNA (RNA) or viral vector on a solid support. Transduction is achieved by adding cells to the immobilized DNA or vector, which can save time and labor and reduce readout variation. Although encouraging, improvements are needed before these methods are widely applied. In reverse transfection approaches, the transfection conditions may need to be modified when different cell lines are used. For RI, only a lentiviral vector has been tested [3], which may not be the best choice considering its stability. In this report, we describe an RI protocol based on another frequently used viral vector, an adeno-associated virus (AAV).

AAV is a 20-nm replication-defective virus that infects humans and other primates, yet does not cause any known disease [4]. Vectors derived from AAV are attractive for gene therapy because they transduce both dividing and non-dividing cells and have the ability to mediate long-term transgene expression *in vivo* [5]. To date, at least 11 serotypes (AAV1-11) have been described [6]. Among them, AAV1, AAV2, AAV5, and AAV8 are used more frequently than others in gene therapy studies because of their unique *in vivo* transduction profiles. AAV1 has the highest transduction efficiency in muscle [7], AAV2 has a broad tissue tropism, including muscle, liver, and the retina [8], AAV5 has the highest transduction efficiency in respiratory ducts [9], and AAV8 shows strong liver tropism [10]. AAVs with mosaic capsids represent one strategy for creating new tropisms [11]. These AAV serotypes or variants also display different transduction profiles *in vitro*.

As a non-enveloped icosahedron particle, AAV has distinct characteristics, including considerable resistance to heat, organic solvents, and pH extremes [12]. Moreover, AAV infection causes little cellular toxicity, which is a common consequence of other vectors [13]. It is these advantages that prompted us to attempt to expand the *in vitro* applications of AAVs.

In this study, we established an AAV array in which quantified recombinant AAVs (rAAVs) were coated on the wells of 96-well plates. The medium was then allowed to evaporate, and the coated plates were stored until use. Cells were then added to the prepared plates to achieve gene transduction. Our study provides a convenient high-throughput approach to gene transduction for biological research.

## Results

The AAV plasmid, genomic structure of the helper virus used for rAAV packaging, and electron microscopic assessment.

**Figure 1:**
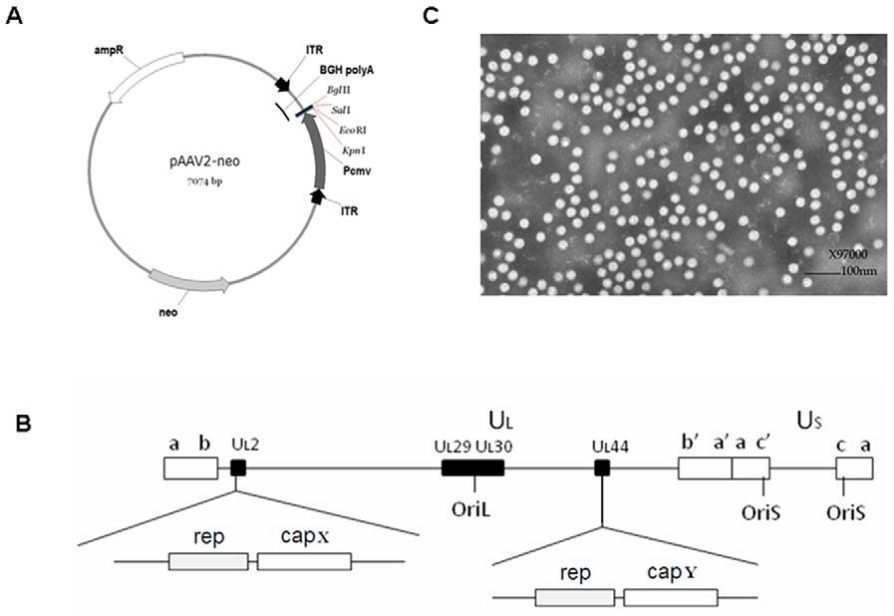
The AAV plasmid, genomic structure of the helper virus used for rAAV packaging, and electron microscopic assessment. A. Map of the AAV plasmid, showing the reporter gene; Gluc or EGFP was inserted between the CMV promoter and polyA region. B. Helper viruses used for packaging of the rAAV with different capsids. UL2 and UL44 were both used for repcap insertion. capX and capY represent capsid genes from the different AAV serotypes. C. Electron microscopic image of rAAV2-Gluc.

AAV vectors harboring reporter genes were constructed by inserting the genes encoding *Gaussia* luciferase (Gluc) or enhanced green fluorescent protein (EGFP) between the cytomegalovirus (CMV) promoter and bovine growth hormone polyA (BGH polyA) in pAAV2neo (Fig. 1A) or pAAV5neo [14]. rAAVs were produced by infecting the AAV vector cell lines with recombinant herpes simplex virus carrying the AAV rep and cap genes (Fig. 1B); purification was achieved as described previously [15]. The purified rAAV particles were somewhat round (20–24 nm in diameter; Fig. 1C). The viral titers of the rAAVs were measured by dot blotting, as described in the Materials and Methods, and diluted to equal titers for coating the plates.

### Optimization of rAAV RI

**Figure 2:**
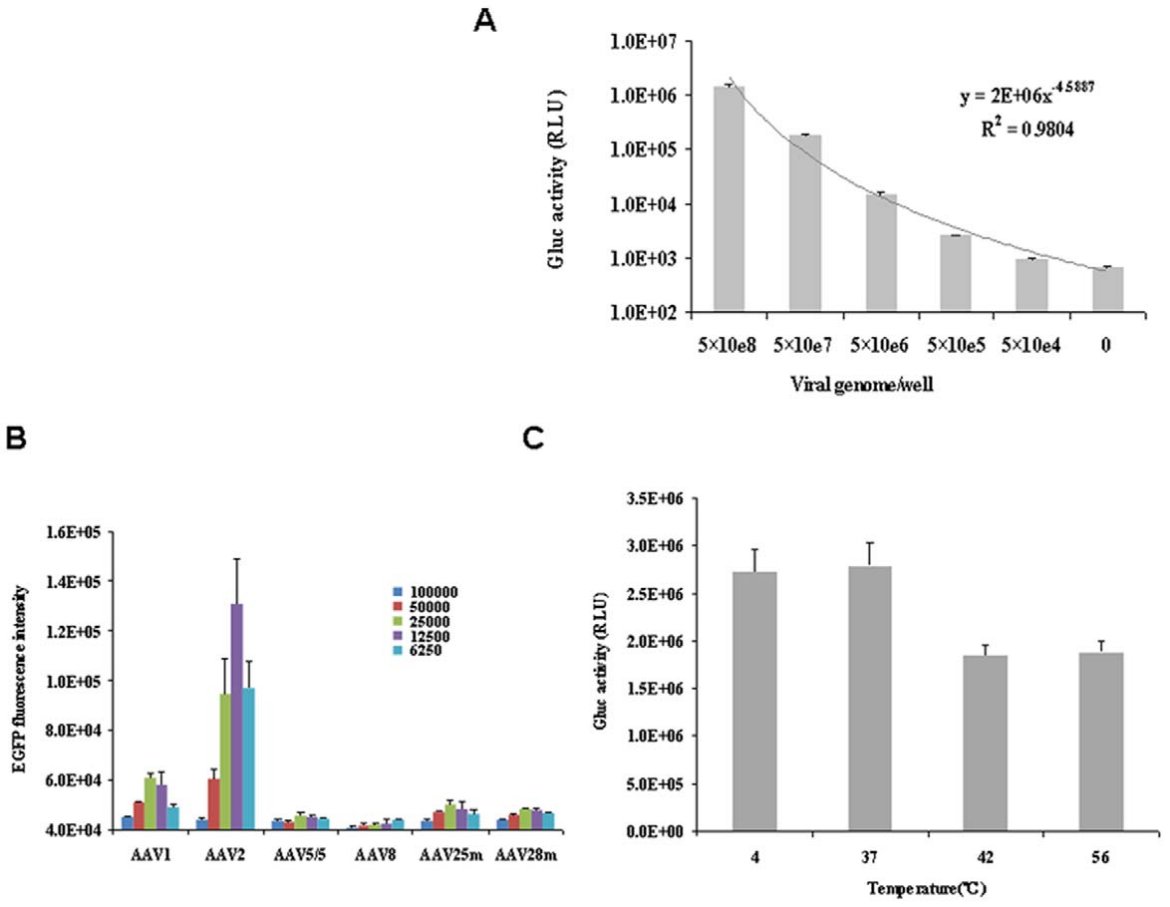
Optimization of rAAV RI. A. Transduction efficiency of different quantities of rAAV2-Gluc using 4×10^4^ BHK21 cells; the transduction efficiency is represented by Gluc activity. B. Optimization of the virus/cell ratio. Different numbers of BHK21 cells were applied to a 96-well plate pre-coated with 5×10^8^ viral genomes/well. The transduction efficiency is represented by the EGFP intensity. C. Temperature stability assessment of rAAV2-Gluc. In total, 5×10^8^ viral genomes of rAAV were applied to each well. The plates were then treated at 4, 37, 42, or 56°C for 24 h, followed by the application of 4×10^4^ BHK21 cells per well. Gluc activity was measured 24 h later.

To determine the optimal amount of rAAV per well, 5×10^4^, 5×10^5^, 5×10^6^, 5×10^7^, and 5×10^8^ viral genomes of rAAV2-Gluc were applied to each well. After drying, 4×10^4^ BHK21 cells were applied to each well. Then, 24 h later, Gluc activity was measured. As shown in Figure 2A, Gluc activity was positively correlated with viral load; as the viral load increased, more Gluc activity was observed. Considering the different infectivity of AAV for different cells, we chose to coat each well with 5×10^8^ viral genomes in each of the remaining experiments.

To determine the optimal number of cells per well, various numbers of BHK21 cells were applied to the wells coated with the same quantity of viral genomes. Then, 48 h later, EGFP fluorescence was assayed using an EnVision Multilabel Plate Reader (Perkin Elmer, Waltham, MA). As shown in Figure 2B, with the exception of AAV8, the highest level of EGFP intensity was achieved by adding either 2.5×10^4^ or 1.25×10^4^ cells per well; thus, 20,000–40,000 was taken as the optimal virus/cell ratio to obtain the highest transduction efficiency. Because the highest level of expression for most AAV serotypes was seen in the wells with 2.5×10^4^ cells, we used this cell density in all subsequent experiments.

To assess the temperature stability of the rAAV2-coated plates, we placed the 96-well plates in incubators set at 37, 42, and 56°C for 24 h before application of the BHK21 cells. A plate stored at 4°C was used as a control. As shown in Figure 2C, no difference in Gluc activity was observed between the control plate stored at 4°C and the plate incubated at 37°C. The level of Gluc activity in the plates incubated at 42 and 56°C showed a 31–33% decrease. However, the total level of Gluc activity was still high, suggesting that the coated rAAVs were resistant to temperatures as high as 56°C for at least 24 h. Taken together, these data demonstrate that rAAV-coated 96-well plates can be used for RI, and that the rAAVs had not lost any infectivity when stored at 37°C for at least 24 h.

### Transduction efficiency of the rAAVs in different cell lines

**Figure 3:**
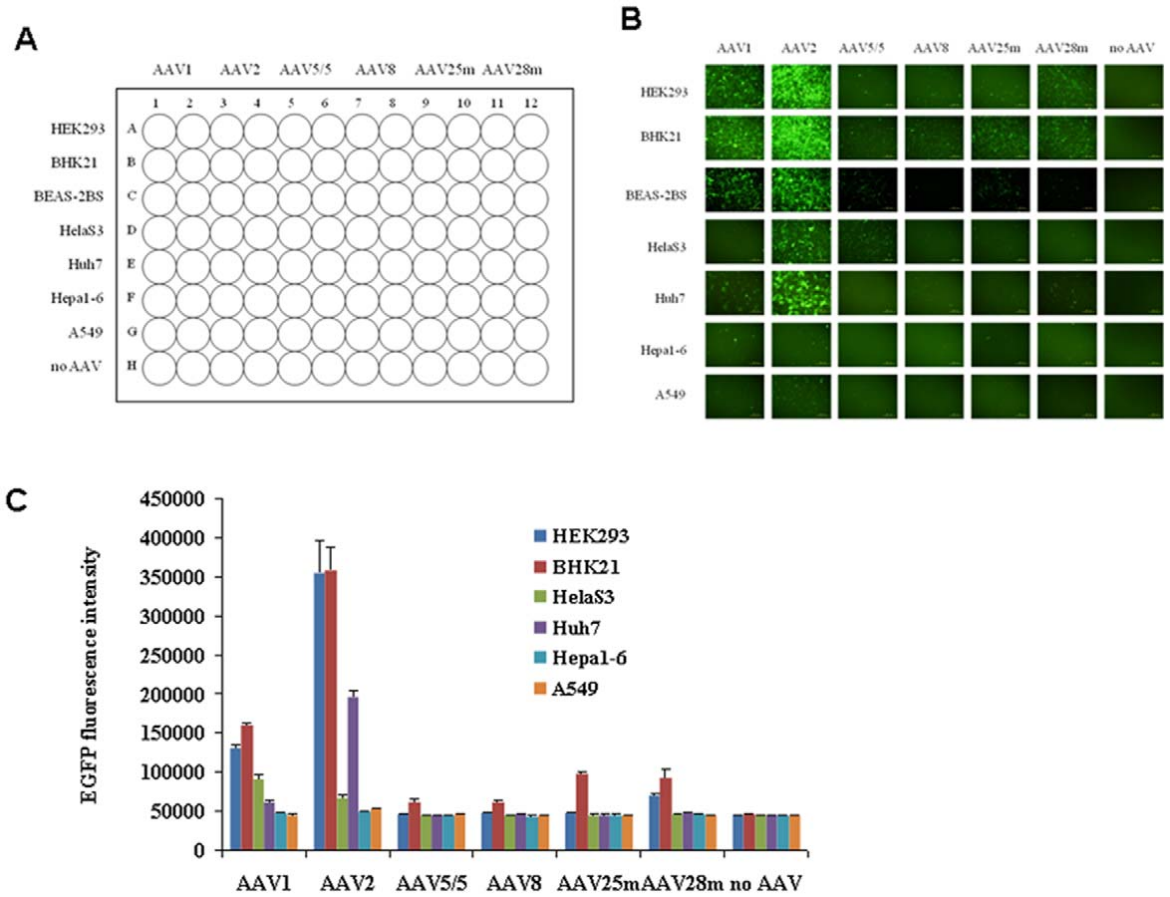
Description of the rAAV RI-based array plate. A. Schematic view of the AAV-based RI array plate. Six types of rAAVs were applied to 96-well plates in two columns, allowing seven types of cells (and a control) to be applied simultaneously to the assay plate. B. Fluorescence microscopic assessment of the infectivity of the rAAVs. C. EGFP intensity summary of Figure 2B.

It has been reported that different rAAV serotypes displayed different transduction efficiencies; thus, we next evaluated the transduction efficiency of the rAAVs rAAV1, rAAV2, rAAV5/5, and rAAV8 using multiple cell lines and the above protocol. Hybrid vectors between AAV2 and 5 (designated rAAV25 m), and AAV2 and AAV8 (designated rAAV28 m) were also included in the test. The cell lines were then applied to each viral line, as shown in Figure 3A. rAAV5/5, rAAV8, rAAV25 m, and rAAV28 m showed low transduction efficiency in these cell lines; conversely, rAAV1 and, especially, rAAV2 showed high transduction efficiency in many cell lines, as assessed by fluorescence microscopy (Fig. 3B). rAAV2 was able to infect HEK293, BHK21, BEAS-2BS, and Huh7 cells with high efficiency, whereas rAAV1 was able to infect HEK293, BHK21, BEAS-2BS, HelaS3, and Huh7 cells with considerable efficiency (Fig. 3B). Among these cell lines, BHK21 was the most susceptible to the rAAVs (Fig. 3B). To quantify our fluorescence microscopic observations, the level of EGFP intensity in each plate was measured using an EnVision Multilabel Plate Reader (Perkin Elmer). As shown in Figure 3C, the data were consistent with those produced by fluorescence microscopy. In conclusion, we found that rAAV2 and rAAV1 were suitable for RI as part of a high-throughput array strategy.

### Response to treatment with sodium butyrate (NaB)

**Figure 4:**
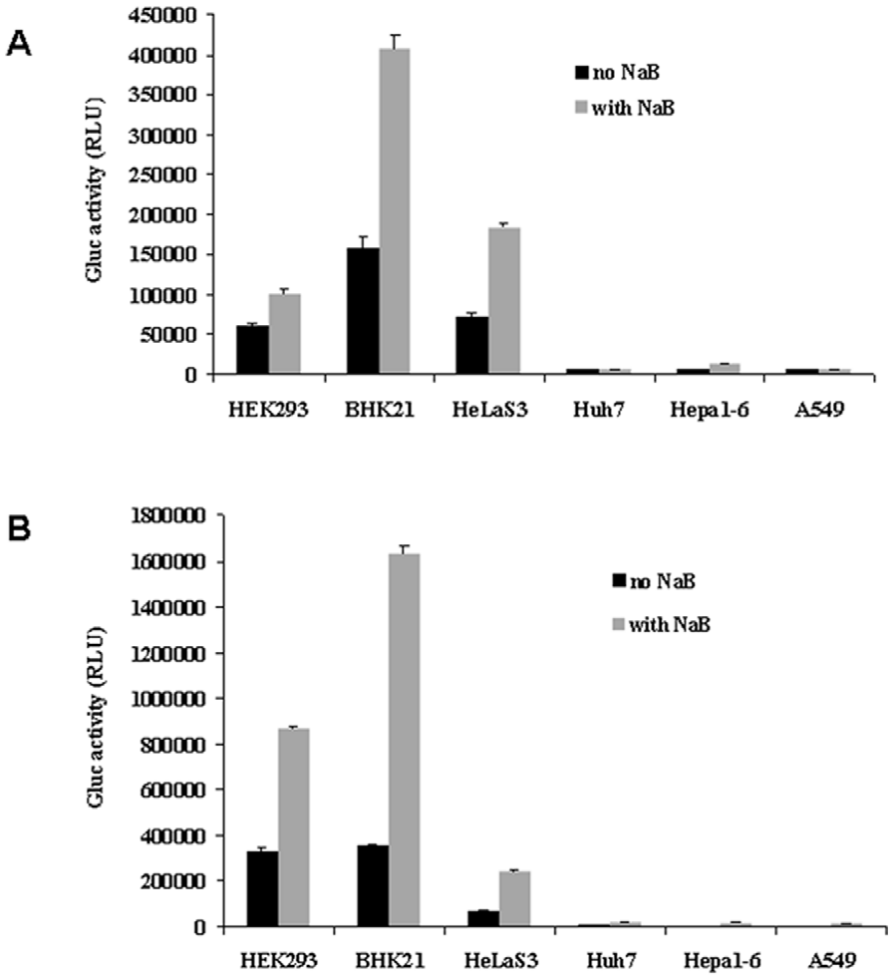
Response to NaB. Black bar, without NaB; gray bar, with NaB. A. AAV1 RI-based assay. B. AAV2 RI-based assay.

We next evaluated the responses of the rAAV-transduced cell lines to NaB, which was used as an interfering factor. NaB is known to enhance gene expression [16]. Cells were applied to wells coated with rAAV1 or rAAV2 in the presence or absence of 10 mM NaB. Then, 24 h later, Gluc activity in the supernatant was measured. Gluc activity in the cells transduced by rAAV1 (Fig. 4A) or rAAV2 (Fig. 4B) was increased significantly, especially for HEK293 and BHK21. Of rAAV2, Gluc activity in BHK21 increased from 3×10^5^ to 1.6×10^6^ relative light units (RLU; Fig. 4B). Of rAAV1, Gluc activity in the BHK21 cells increased, from 1.5×10^5^ to 4×10^5^ RLU (Fig. 4A). In conclusion, our data show that our system can be used to monitor the response to a modulating factor with substantial sensitivity.

## Discussion

Current *in vitro* gene transfer approaches, based on liposomes and cationic polymers, are both efficient and convenient. However, they sometimes show relatively low reproducibility due to various factors in the transfection procedure. To reduce readout variation, the order of addition of DNA and adherent cells has been reversed relative to conventional transfection methods (i.e., the DNA is printed on a glass slide before addition of the adherent cells). The advantages of reverse transfection [17] procedures, compared with conventional transfection methods, include reduced labor, the use of fewer materials, and the ability to perform high-throughput screening. However, the efficiency of reverse transfection varies depending on the cell type being used, and this, in turn, limits its use in high-throughput screening. The retroviral microarray-based platform created by Carbone et al. [3] showed an enabling tool for functional genomics and drug discovery. In the present study, we developed a novel gene transduction approach by pre-coating rAAVs on a cell culture plate and allowing them to dry until use. Our data show that the rAAVs were resistant to temperature changes. Furthermore, rAAVs coated on plates have been stored at 4°C for more than half a year without an apparent loss of infectivity (data not shown).

AAV vectors have been broadly used in gene therapy studies, yet their application to *in vitro* high-throughput transduction is limited. In the present study, the transduction efficiency of different rAAV serotypes or variants was evaluated in HEK293, BHK21, BEAS-2BS, HelaS3, Huh7, Hepa1-6, and A549 cells. Among the AAV vectors tested, rAAV1 and rAAV2 displayed the most promising results when used for RI.

To our knowledge, this is the first report describing the RI of AAV vectors. The data in the present study suggest that our AAV RI method can be used to create “infection-ready” AAV arrays for high-throughput biological assays. This type of array can be used to test the response of cells to certain interfering factors, as exemplified in this study. RNAi screening can also be achieved if an AAV-based shRNA library is applied to the array. Additionally, microRNA profiles of cell lines can be established if microRNA target sequences linked with reporter genes are applied; this may become an alternative and supplemental tool for microarray analysis.

## Materials and Methods

### Cell culture

BHK21, HEK293, BEAS-2BS, HeLaS3, Huh7, Hepa1-6, and A549 cells were maintained as monolayer cultures in Dulbecco's Modified Eagle's Medium (DMEM) containing 10% fetal calf serum, 100 µg/mL penicillin, and 100 U/mL streptomycin, as recommended by the manufacturer (GIBCO, Gaithersburg, MD).

### Plasmid construction

pAAV2neo was constructed by inserting a fragment containing two inverted terminal repeats (ITRs) from AAV2, the CMV promoter, multi-cloning sites, and BGH polyA into pSV2neo [18]. pAAV2neo-Gluc and pAAV2neo-EGFP were constructed by inserting the genes encoding Gluc and EGFP between the CMV promoter and BGH polyA. pAAV5neo has a similar structure to pAAV2neo, except that it carries ITRs from AAV5 rather than from AAV2. pAAV5neo-EGFP was constructed by inserting EGFP between the CMV promoter and BGH polyA in pAAV5neo.

### rAAV vector cell lines

rAAV vector cell lines were obtained by transfecting pAAV2neo-Gluc, pAAV2neo-EGFP, or pAAV5neo-EGFP [14] into BHK21 cells, which were then cultured in the presence of 800 µg/mL G418 for 2 weeks.

### Preparation of competent helper virus

Recombinant herpes simplex virus type 1 carrying the AAV rep and cap genes (HSV1-RC) was used to provide helper functions for AAV vector packaging. rHSV1-UL2/rep2cap1, rHSV1-UL2/rep2cap2, and rHSV1-UL2/rep2cap8 were used to package the rAAV1, rAAV2, and rAAV8 (containing the ITR of serotype 2 and corresponding capsid of serotypes 1, 2, and 8) vectors, respectively; rHSV1-UL2/rep5cap5 was used for rAAV5/5 (containing the ITR of serotype 5 and capsid of serotype 5). rHSV1-UL2/rep2cap2-UL44/rep5cap5 and rHSV1-UL2/rep2cap2-UL44/rep2cap8 were used for rAAV25 m (containing the ITR of serotype 2 and mosaic capsid of serotypes 2 and 5) and rAAV28 m (containing the ITR of serotype 2 and mosaic capsid of serotypes 2 and 8), respectively.

### rAAV production

rAAVs were produced by infecting the AAV vector cell lines with HSV1-RC at an MOI of 1–5. After 48–72 h, the infected cells were harvested and purified, as described previously [15], [19]. The rAAV titer was determined by dot blotting using a digoxin-labeled CMV promoter fragment as the probe.

### Transmission electron microscopy

Purified rAAVs were observed by transmission electron microscopy (TECNAI 12, FEI, Blackwood, NJ) with an acceleration voltage of 80 kV, as reported by Chen et al. [20].

### Preparation of the rAAV-coated plates

To prepare rAAV-coated plates, 20 µL/well of rAAVs with defined titers were added to a 96-well plate and placed under airflow overnight in a tissue culture hood to evaporate all liquid. The plate was stored at 4°C until use.

### RI protocol

Cells were digested with 0.25% trypsin (Invitrogen, Carlsbad, CA) to create a single-cell suspension. An equal volume of the suspension (200 µL/well) was applied to the rAAV-coated plates and cultured at 37°C in an incubator under 5% CO_2_ for 24–48 h.

### Gluc activity assay

The Gluc activity level was measured as described in the *Gaussia* luciferase assay kit (New England Biolabs, Ipswich, MA) by adding 50 µL of substrate solution to 20 µL of the supernatant or cell lysate, followed by determination of the RLU using a luminometer.

### Analysis of EGFP expression

EGFP expression was assayed by fluorescence microscopy (Olympus DP70, BH2-RFL-T3). The EGFP intensities in the 96-well plates were measured using an EnVision Multilabel Plate Reader (Perkin Elmer).
